# Cytochrome *bd* promotes *Escherichia coli* biofilm antibiotic tolerance by regulating accumulation of noxious chemicals

**DOI:** 10.1038/s41522-021-00210-x

**Published:** 2021-04-16

**Authors:** Connor J. Beebout, Levy A. Sominsky, Allison R. Eberly, Gerald T. Van Horn, Maria Hadjifrangiskou

**Affiliations:** 1 Department of Pathology, Microbiology, and Immunology, Vanderbilt University Medical Center, Nashville, TN USA; 2 Vanderbilt University, Nashville, TN USA; 3 Vanderbilt Institute for Infection, Immunology, and Inflammation, Vanderbilt University Medical Center, Nashville, TN USA; 4 Present Address: Division of Clinical Microbiology, Department of Laboratory Medicine and Pathology, Mayo Clinic, Rochester, MN USA

**Keywords:** Biofilms, Antimicrobials

## Abstract

Nutrient gradients in biofilms cause bacteria to organize into metabolically versatile communities capable of withstanding threats from external agents including bacteriophages, phagocytes, and antibiotics. We previously determined that oxygen availability spatially organizes respiration in uropathogenic *Escherichia coli* biofilms, and that the high-affinity respiratory quinol oxidase cytochrome *bd* is necessary for extracellular matrix production and biofilm development. In this study we investigate the physiologic consequences of cytochrome *bd* deficiency in biofilms and determine that loss of cytochrome *bd* induces a biofilm-specific increase in expression of general diffusion porins, leading to elevated outer membrane permeability. In addition, loss of cytochrome *bd* impedes the proton mediated efflux of noxious chemicals by diminishing respiratory flux. As a result, loss of cytochrome *bd* enhances cellular accumulation of noxious chemicals and increases biofilm susceptibility to antibiotics. These results identify an undescribed link between *E. coli* biofilm respiration and stress tolerance, while suggesting the possibility of inhibiting cytochrome *bd* as an antibiofilm therapeutic approach.

## Introduction

Biofilms are multicellular bacterial communities commonly encountered in the environment and during infection. Because bacteria in biofilms are intrinsically resistant to a variety of stressors, including antibiotics, phagocytes, and bacteriophages, the ability to form biofilms is a critical bacterial survival strategy^1–7^. Accordingly, the vast majority of bacteria in the environment and in the human body—up to 80 percent according to recent estimates^8^—are believed to exist in the biofilm state. Due to the stress tolerance of biofilms and the lack of biofilm-specific therapies, a biofilm associated infection typically necessitates chronic suppressive antibiotic treatment or surgical removal of the infected material^9^. Improvements in antibiofilm therapeutics are critically needed and would greatly advance our ability to reduce infection burden.

Within biofilms, bacteria consume or alter chemicals as they diffuse through the community, thus generating a variety of nutrient gradients that ensure individual bacteria are exposed to highly variable local environmental conditions^3^. This environmental heterogeneity induces bacteria to differentiate into metabolically distinct, and oftentimes cooperative, subpopulations, which enhance the overall resilience and versatility of the community^2,3,10–12^. We previously hypothesized that the presence of oxygen gradients in biofilms would generate differentially respiring subpopulations, each of which uniquely contributes to biofilm development. Indeed, our work with uropathogenic *E. coli* (UPEC)—the primary cause of urinary tract infections and one of the most common human bacterial pathogens^13–15^—indicates that differential oxygen availability across biofilm regions leads to heterogenous expression of respiratory enzymes, with the aerobic quinol oxidases being the most abundantly expressed^16^.

*E. coli* is a facultative anaerobe that encodes a modular electron transport chain containing a multitude of interchangeable dehydrogenases, quinol electron carriers, and terminal oxidases/reductases^17,18^. This architecture provides *E. coli* an enormous degree of metabolic flexibility, allowing bacteria to colonize diverse niches. Despite being a facultative anaerobe, previous studies establish that UPEC requires aerobic respiration during infection and to form biofilms^10,16,19–23^. During bladder infection, UPEC consumes amino acids, which feed into the TCA cycle to energize the aerobic respiratory chain^19,20,22^. UPEC encodes three aerobic respiratory quinol oxidases: one proton pumping heme-copper oxidase, cytochrome *bo*, and two non-proton pumping *bd*-type oxidases, cytochrome *bd* and cytochrome *bd*_2_^17,24^. Cytochrome *bo* is a low oxygen affinity quinol oxidase transcriptionally and biochemically optimized for use under atmospheric oxygen tensions^25–27^. By contrast, the *bd*-type oxidases have high oxygen affinity and are optimized for use under low oxygen tensions^25–27^. Importantly, the *bd*-type oxidases also provide resistance against oxidative and nitrosative stress, suggesting these enzymes may play a critical role in enhancing stress tolerance under microaerobic conditions encountered in biofilms and the urinary tract^28–30^.

Given the importance of aerobic respiration to UPEC pathogenesis and biofilm formation, as well as the oxygen regulated transcriptional control of quinol oxidases, we previously investigated the spatial distribution of respiration in biofilms^16^. In agreement with studies in *Pseudomonas aeruginosa*, we determined that expression of the two most abundant oxidases, cytochrome *bd* and *bo*, is inversely correlated in the community along the biofilm oxygen gradient, suggesting *E. coli* biofilms contain differentially respiring subpopulations^16,31^. We then sought to disentangle the contributions of each quinol oxidase to biofilm physiology. Surprisingly, despite robust expression of all three aerobic quinol oxidases, only loss of cytochrome *bd* has any significant effect on biofilm development. Cytochrome *bd* deficiency induces severe architectural disturbances in biofilms and reduces their ability to prevent external stressors from entering the biomass^16^. Deletion of the locus that encodes cytochrome *bd* leads to upregulation of the low-affinity oxidase cytochrome *bo* and impairs biofilm development without compromising ATP levels^16^. This study established the presence of differentially respiring subpopulations in *E. coli* biofilms, and argues respiratory heterogeneity is a fundamental contributor to biofilm physiology.

In this work we aimed to determine how cytochrome *bd* expressing biofilm subpopulations contribute to *E. coli* biofilm physiology. To do so, we interrogated and compared the cellular physiology of cytochrome *bd*-deficient cells in the planktonic and biofilm state. We determine that loss of cytochrome *bd* increases the abundance of multiple outer membrane proteins in biofilm cells, including general diffusion porins responsible for antibiotic uptake. Consequently, cytochrome *bd*-deficient biofilm cells have increased outer membrane permeability and more readily take up noxious chemicals from the environment. In addition to enhancing cellular uptake of noxious chemicals, loss of cytochrome *bd* impairs their efflux by impeding the proton dependent activity of resistance-nodulation-division (RND) efflux pumps and possibly other tripartite export proteins. As a result, loss of cytochrome *bd* increases biofilm susceptibility to multiple clinically relevant antibiotics. Interestingly, this increased sensitivity is a biofilm-specific phenomenon, as deletion of cytochrome *bd* has no effect on antibiotic susceptibility in planktonic cells. This study reveals a previously undescribed link between respiration and biofilm stress tolerance in *E. coli* and suggests the possibility of inhibiting cytochrome *bd* as a therapeutic strategy for preventing and treating urinary tract infections.

## Results

### Loss of cytochrome *bd* increases biofilm antibiotic sensitivity

We previously determined that uropathogenic *Escherichia coli* (UPEC) exhibits marked respiratory heterogeneity in biofilms, and that loss of cytochrome *bd*—but not other respiratory quinol oxidases—induces significant disruptions to biofilm development^16^. Furthermore, we demonstrated that these disruptions are solely attributable to cytochrome *bd* deficiency, as extrachromosomal complementation of ∆*cydAB* with a plasmid encoding the *cydABX* operon under native transcriptional control fully rescues the observed biofilm deficits^16^. Based on these observations, we hypothesized that cytochrome *bd* is necessary for the formation of metabolically versatile biofilm communities capable of withstanding antibiotics and other external stressors. To test this, we first evaluated the effects of antibiotics on biofilms formed by the well-characterized uropathogenic *Escherichia coli* cystitis isolate UTI89 and an isogenic mutant strain lacking cytochrome *bd* (∆*cydAB*)^16^.

**Fig. 1:**
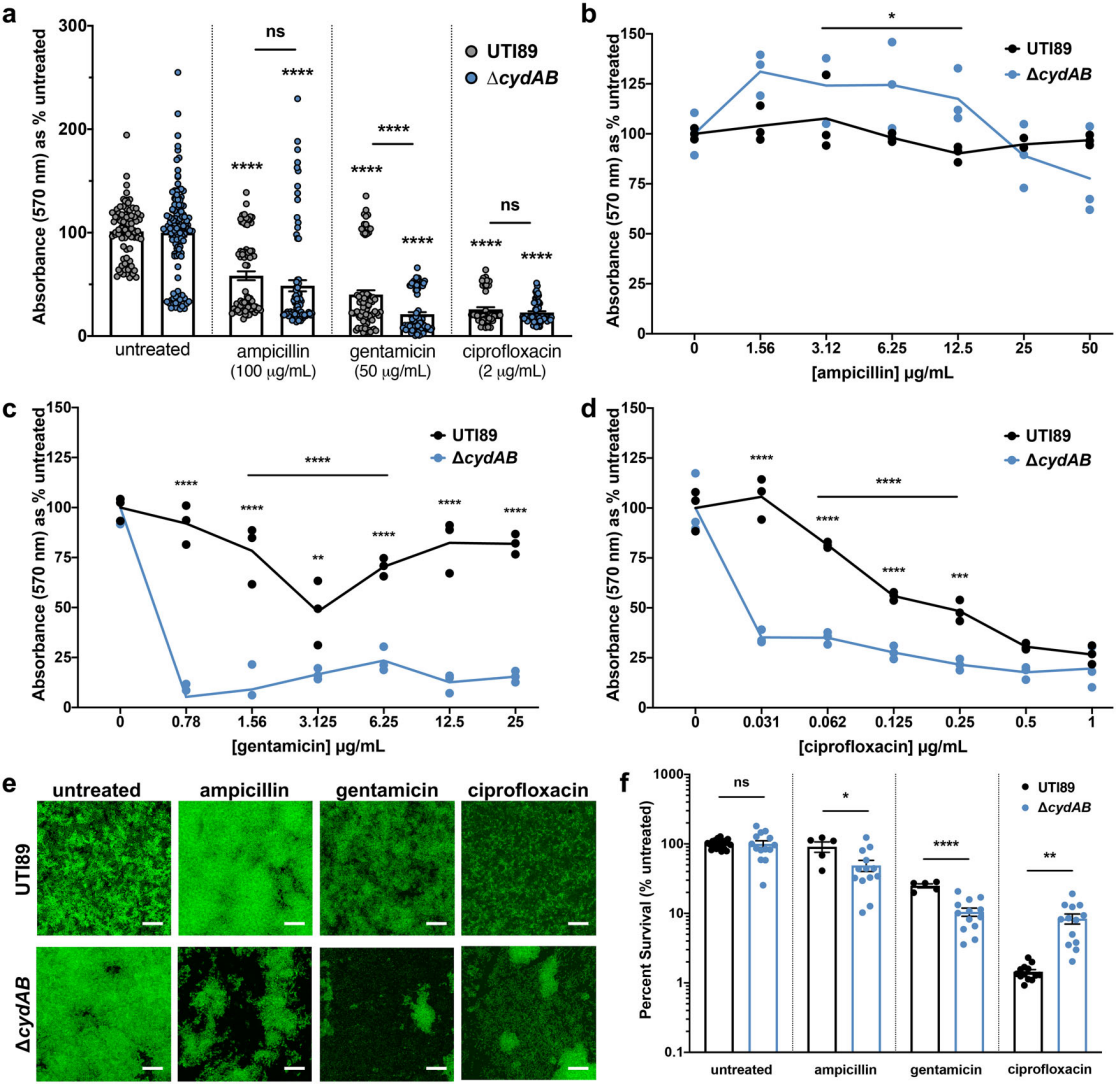
Loss of cytochrome *bd* increases biofilm antibiotic sensitivity. **a** PVC-associated air-liquid interface biofilms were grown for 48 h, treated with antibiotics for 72 h, and biomass was quantified using the crystal violet assay. Biofilm biomass was quantified for wild-type UTI89 and isogenic cytochrome *bd*-deficient strain ∆*cydAB*. Data were normalized to the untreated control of each strain. The mean absorbance value in each treatment group was compared to the untreated control of the same strain (asterisks), and to the mean value of the other strain in the same treatment group (horizontal line with asterisks) using a two-tailed Welch’s *t*-test. Data represent at least three biological replicates with at least eight technical replicates each. Each dot represents an independent well. **b**–**d** Dose response curves depicting the total biomass of biofilms after treatment with decreasing concentrations of ampicillin (**b**), gentamicin (**c**), or ciprofloxacin (**d**). Data were analyzed using a two-way ANOVA to evaluate overall differences between strain across the range of concentrations tested (horizontal line with asterisks), with multiple comparisons used to evaluate differences in mean at each concentration (asterisks). Data represent three biological replicates with three technical replicates each. Each dot represents the mean value of a biological replicate. Solid lines connect mean values at each concentration. **e** Representative images of antibiotic treated biofilms stained with STYO9 and imaged by confocal laser scanning microscopy. At least five images were acquired along the air-liquid interface of three biological replicates. Scale bar is 20 µm. **f** Survival of bacteria in colony biofilms after antibiotic treatment. Colony biofilms were grown on YESCA agar for 72 h, and biofilms were transferred to a new plate with or without antibiotics. After 24 h of antibiotic treatment, biofilms were homogenized and plated to enumerate CFUs. Mean values in each treatment group were statistically compared using a two-tailed unpaired *t*-test. Data are representative of at least five biological replicates. Each dot represents a biological replicate. For all graphs, data are presented as mean ± SEM. *p < 0.05, **p < 0.01, ***p < 0.001, ****p < 0.0001.

A recent meta-analysis demonstrates that measuring biofilm antimicrobial susceptibility using a single method or a single drug concentration is often inadequate due to a high degree of variability between methods^32^. As such, we sought to investigate the susceptibility of ∆*cydAB* biofilms antibiotic susceptibility across a range of conditions. First, we grew polyvinyl chloride (PVC)-associated biofilms for 48 h, treated with a panel of antibiotics for another 72 h, and measured overall biofilm abundance by the crystal violet assay^33^. Treatment of wild-type biofilms with supralethal doses of β-lactams (ampicillin), aminoglycosides (gentamicin), or fluoroquinolones (ciprofloxacin) led to a 40–75% reduction in total biomass but did not eradicate the biofilm, highlighting the resilience of biofilms in the face of our current therapeutic strategies (Fig. 1a). After normalizing biomass to the untreated control of each strain, we determined both strains have similar relative reductions in biomass after treatment with β-lactams or fluoroquinolones, but ∆*cydAB* biofilms are significantly more susceptible to aminoglycosides than the parental strain (Fig. 1a).

To more thoroughly define biofilm sensitivity to antibiotics and more closely approximate antibiotic concentrations encountered by bacteria under clinically relevant conditions, we grew PVC-associated biofilms as previously described and treated with decreasing concentrations of antibiotics (Fig. 1b–d). Interestingly, at lower β-lactam concentrations we observe minimal effect on total biomass, and ∆*cydAB* biofilms exhibit a small, but statistically significant increase in biomass relative to wild-type (Fig. 1b). By contrast, ∆*cydAB* biofilms are significantly more sensitive to both aminoglycosides and fluoroquinolones across the range of concentrations tested (Fig. 1c, d). This effect was most pronounced at lower antibiotic concentrations similar to serum antibiotic concentrations achieved clinically.

Although the crystal violet assay represents a convenient method of assessing biofilm biomass, it does not provide meaningful insights into biofilm architecture or physiology^34^. To characterize the structural effect of antibiotics on Δ*cydAB* biofilms, we grew biofilms on PVC coverslips, treated with antibiotics as described above, and imaged the biofilms using confocal laser scanning microscopy (Fig. 1e). As expected, antibiotic treatment had relatively minor effects on the structural characteristics of wild-type biofilms (Fig. 1e). Whereas β-lactam treatment led to topographic changes in wild-type biofilms without affecting the apparent density of cells, aminoglycoside or fluoroquinolone treatment had minimal effect (Fig. 1e). By contrast, treatment with all three classes of antibiotics led to widespread structural disruption of ∆*cydAB* biofilms and reduced cell density (Fig. 1e), grossly consistent with the reductions in biomass observed by crystal violet assays.

The above data indicate that antibiotic treatment induces a more significant loss of biomass in ∆*cydAB* biofilms as compared to those biofilms formed by the wild-type strain. To determine whether the antibiotic-induced biomass reductions are caused by increased cell death, we quantified percent survival of biofilm cells after 24 h of antibiotic treatment in a colony biofilm model^35^. Treatment of wild-type biofilms with β-lactams had no significant effect on biofilm CFUs, whereas treatment with aminoglycosides or fluoroquinolones led to significant reductions in CFUs per biofilm (Fig. 1f). When compared to wild-type, ∆*cydAB* biofilms had significantly reduced cell survival after treatment with β-lactams (91 and 49 percent survival in UTI89 and ∆*cydAB*, respectively) and aminoglycosides (25 and 10% survival in UTI89 and ∆*cydAB*, respectively), indicating ∆*cydAB* biofilm cells are more sensitive to antibiotic-induced cell death (Fig. 1f). By contrast, ∆*cydAB* biofilm cells are somewhat less sensitive to fluoroquinolone treatment than wild-type (2 and 8 percent survival in UTI89 and ∆*cydAB*, respectively) (Fig. 1f). In total, despite some expected variability between experimental approaches^32^, these data demonstrate that loss of cytochrome *bd* renders biofilms more susceptible to the clinically important β-lactam, aminoglycoside, and fluoroquinolone classes of antibiotics.

### Cytochrome *bd* does not affect planktonic susceptibility to antibiotics

**Fig. 2:**
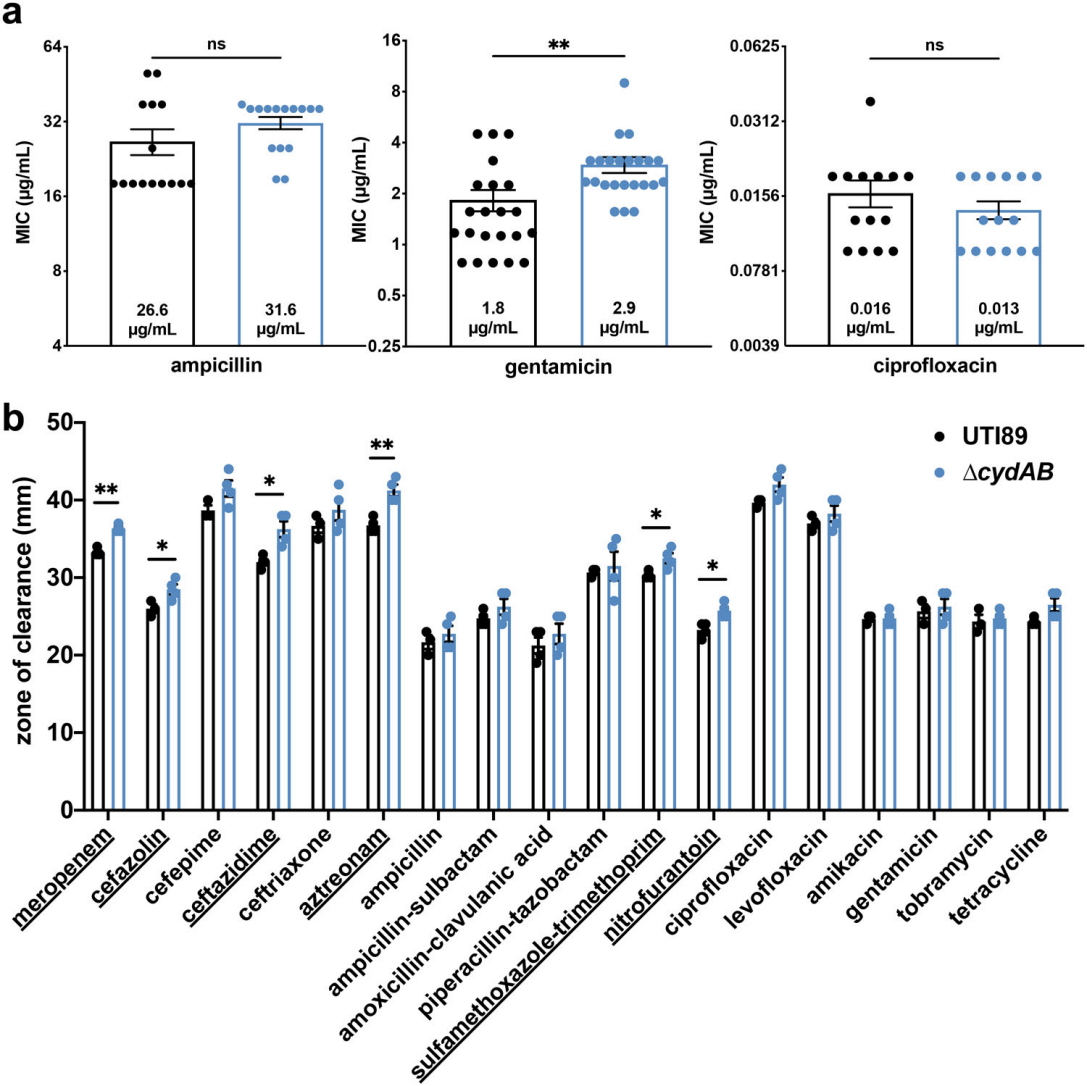
Cytochrome *bd* has minimal effect on planktonic antibiotic susceptibility. **a** Graph depicting minimum inhibitory concentration (MIC) for ampicillin, gentamicin, and ciprofloxacin. Data are representative of at least six biological replicates. Data were analyzed using a two-tailed unpaired *t*-test. **b** Graph depicting the zone of inhibition from disk diffusion assays. Data are representative of at least three biological replicates. Each dot represents a biological replicate. Mean values were statistically compared using two-tailed Welch’s *t*-test. Underlined antibiotics attained statistical significance. For all panels, data are presented as mean ± SEM. *p < 0.05, **p < 0.01, ***p < 0.001, ****p < 0.0001.

Our results thus far indicate that cytochrome *bd* affects the ability of biofilms to withstand antibiotics. To determine whether cytochrome *bd* influences antibiotic sensitivity in the planktonic state, we performed broth microdilution assays to measure the minimum inhibitory concentration (MIC) of ampicillin, gentamicin, and ciprofloxacin against each strain (Fig. 2a). Surprisingly, we observe no significant differences in MIC between strains for ampicillin and ciprofloxacin, and a small increase in MIC for ∆*cydAB* for gentamicin (1.8 and 2.9 µg/mL for UTI89 and ∆*cydAB*, respectively). These MIC values are similar to previously reported values for UTI89, although we observe a somewhat elevated MIC for ampicillin as compared to previous studies^36^. Next, to assess antibiotic sensitivity across a range of clinically relevant antibiotics, we performed disk diffusion assays according to Clinical & Laboratory Standards Institute (CLSI) guidelines and procedures followed by the clinical microbiology laboratory at Vanderbilt University Medical Center^37^. These analyses revealed that ∆*cydAB* had a slightly larger zone of inhibition for most antibiotics tested (median percent difference: 6.5 percent; range: 0–13 percent) (Fig. 2b and Supplementary Table 1). ∆*cydAB* had a significantly larger zone of inhibition after treatment with six antibiotics: meropenem, cefazolin, ceftazidime, aztreonam, sulfamethoxazole-trimethoprim, and nitrofurantoin (Fig. 2b, underlined). Of note, two of these antibiotics—sulfamethoxazole-trimethoprim and nitrofurantoin—are first line treatments for urinary tract infections^38^. Interestingly, we do not observe differences in sensitivity to gentamicin using disk diffusion assays despite the observed increase in MIC, highlighting the effects of distinct growth conditions (static liquid culture versus solid agar surface) on metabolism and antibiotic susceptibility. Although clinical guidelines and CLSI breakpoints would deem both strains equally susceptible to antibiotics (Supplementary Table 1)^37^, these analyses reveal a small but consistent trend toward increased antibiotic susceptibility in ∆*cydAB*, which may be indicative of metabolic derangements also present in the planktonic state.

**Fig. 3:**
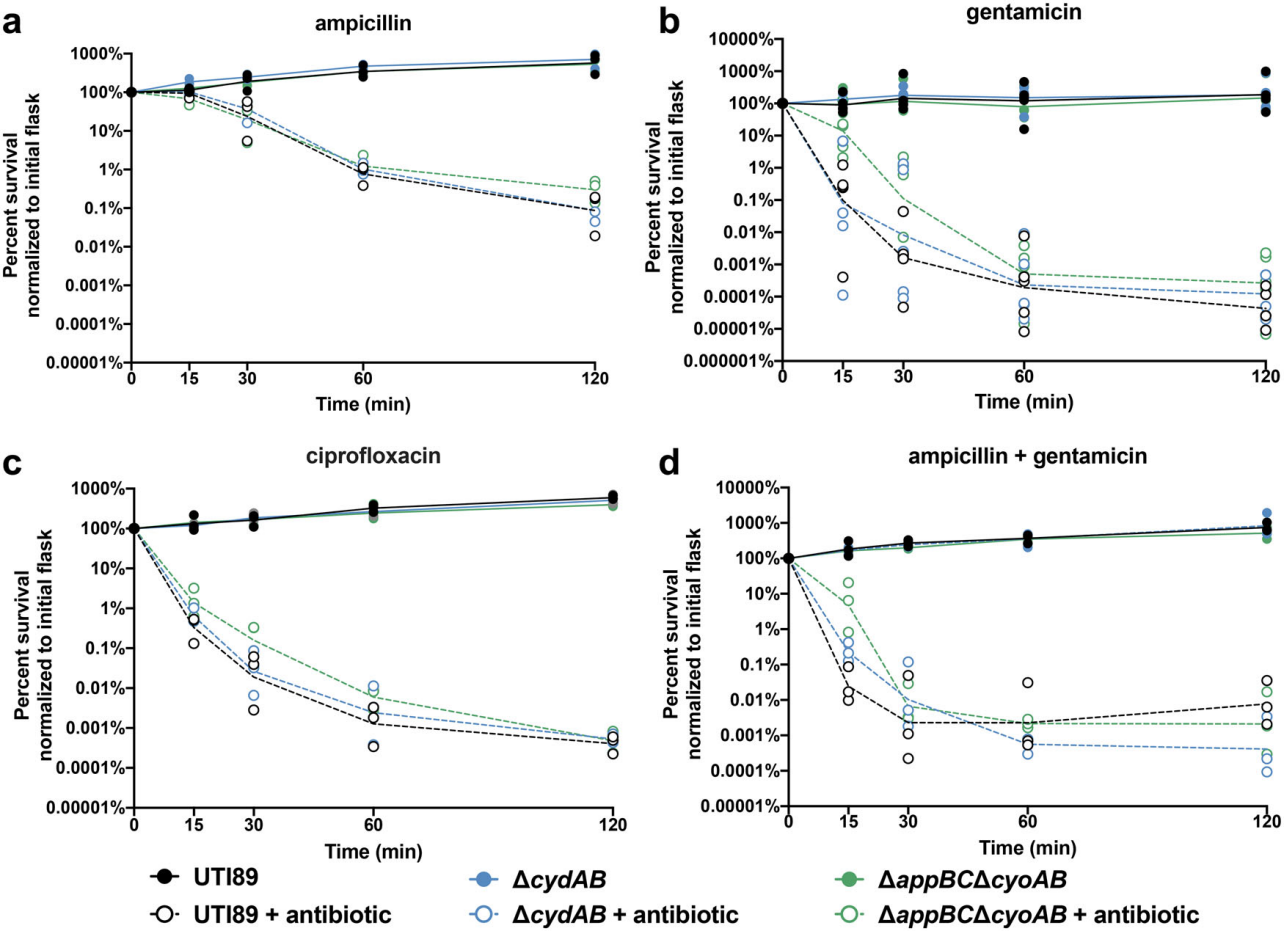
Cytochrome *bd* does not affect the rate of antibiotic-induced cell death in planktonic cells. **a**–**d** Time kill kinetics assays were performed to evaluate the susceptibility of UTI89 (black), ∆*cydAB* (blue), and ∆*appBC*∆*cyoAB* (green) to ampicillin (**a**), gentamicin (**b**), ciprofloxacin (**c**), or a combination of ampicillin and gentamicin (**d**) at 5x MIC. Data were analyzed by a two-way ANOVA with Tukey’s multiple comparisons test and determined to have no significant differences between strains. Data are representative of at least three biological replicates. Lines connect geometric mean at each time point. Each dot represents a biological replicate.

To determine whether the observed changes in antibiotic sensitivity are physiologically relevant, we performed time kill kinetics assays to measure the rate of antibiotic-induced cell death in planktonic cultures (Fig. 3). Cultures were grown to mid-logarithmic phase, split in two, and one flask was treated with antibiotics at 5× the minimal inhibitory concentration. The survival curves were analyzed using a two-way ANOVA to statistically compare the rate of antibiotic-induced cell death between strains over time. To fully evaluate the potential role of cytochrome *bd* in planktonic antibiotic sensitivity, these assays were performed on wild-type, ∆*cydAB*, and a strain that encodes cytochrome *bd* as its sole aerobic quinol oxidase (∆*appBC*∆*cyoAB*). These assays revealed no statistically significant differences in the rate of antibiotic killing between strains after treatment with β-lactams, aminoglycosides, fluoroquinolones, or a clinically relevant synergistic combination of β-lactams and aminoglycosides (Fig. 3a–d). We observe a high degree of variability in survival during the early time points (15–30 min) of cultures treated with aminoglycosides as compared with other antibiotics, raising the possibility that there is heterogeneous early response to aminoglycoside treatment (Fig. 3b, d). These results may reflect differences in antibiotic uptake or efficacy resulting from alterations in the electron transport chain composition and proton motive force. In total, these results indicate that, despite small changes in antibiotic susceptibility observed between strains, loss of cytochrome *bd* has no significant or clinically relevant effect on antibiotic susceptibility in the planktonic state. These results are in agreement with recent work in K-12 *E. coli* demonstrating that loss of *cydB* has no discernible effect on planktonic sensitivity to reactive oxygen species or aminoglycosides^39^. In combination with previous data, these results demonstrate that loss of cytochrome *bd* specifically increases bacterial susceptibility to antibiotics in the biofilm state.

### Loss of cytochrome *bd* alters the outer membrane of biofilm cells

Our data indicate that loss of cytochrome *bd* increases biofilm sensitivity to antibiotics without significantly affecting planktonic sensitivity. In principle, such a biofilm-specific effect could be caused by changes to the extracellular matrix, outer membrane, or cellular metabolism. In our previous work, we reported that ∆*cydAB* biofilms have changes to the abundance, composition, and organization of the extracellular matrix, suggesting that changes in the extracellular matrix may play a role in antibiotic tolerance by influencing community organization and modulating antibiotic sequestration^16^. In this study, we investigate how cytochrome *bd* impacts biofilm antibiotic tolerance by influencing the outer membrane and cellular energetics.

**Fig. 4:**
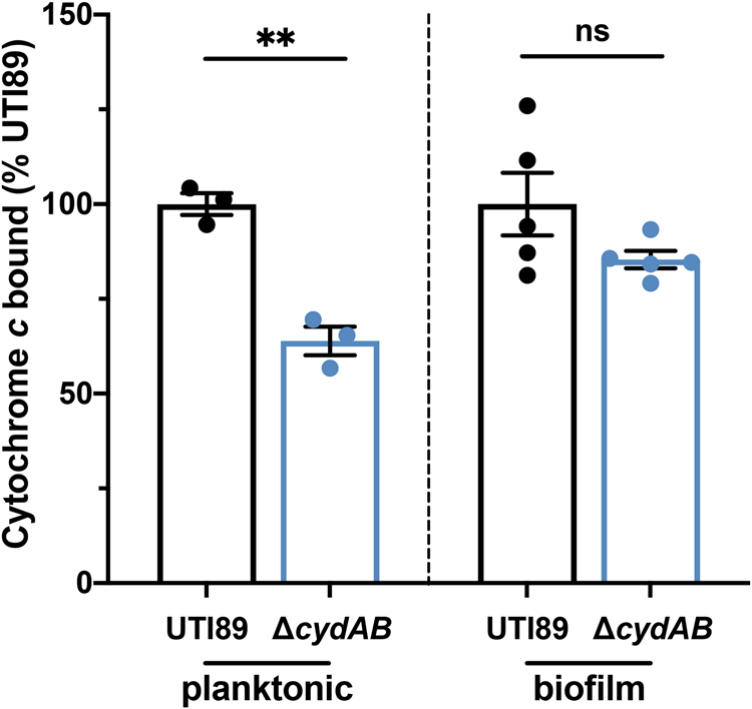
Outer membrane charge is unaffected in ∆cydAB biofilm cells. Outer membrane charge was investigated by measuring the binding of cationic cytochrome *c* to planktonic and biofilm cells. Data were analyzed by a two-tailed unpaired *t*-test. Data are representative of at least three biological replicates, and are presented as mean ± SEM. Each dot represents a biological replicate. *p < 0.05, **p < 0.01, ***p < 0.001, ****p < 0.0001.

Prior to import into the cell, antibiotics must interact with and traverse the negatively charged outer membrane. This step is particularly important for charged antibiotics such as cationic aminoglycosides. As such, one possible explanation for the alterations in antibiotic efficacy is that ∆*cydAB* cells have changes to the charge of their outer membrane with consequent effects on antibiotic import. To test this possibility, we measured the interaction of equine cytochrome *c* with the outer membrane of biofilm and planktonic cells^40^. Cytochrome *c* is a polycationic molecule known to interact electrostatically with the negatively charged bacterial cell envelope^40^. Quantifying the binding of cytochrome *c* to the cell envelope can be used as a proxy for determining the relative charge of the cell envelope, as described previously^40^. In planktonic cells, we observe less cytochrome *c* binding to the outer membrane (63 percent of wild-type), suggesting wild-type cells have a more negatively charged outer membrane than ∆*cydAB* (Fig. 4). However, in biofilm cells, no significant difference in cytochrome *c* binding is observed between wild-type and ∆*cydAB* (Fig. 4) suggesting the outer membrane has a similar charge in both strains. These results indicate that changes to outer membrane charge cannot explain the altered antibiotic sensitivity of ∆*cydAB* biofilm cells.

**Fig. 5:**
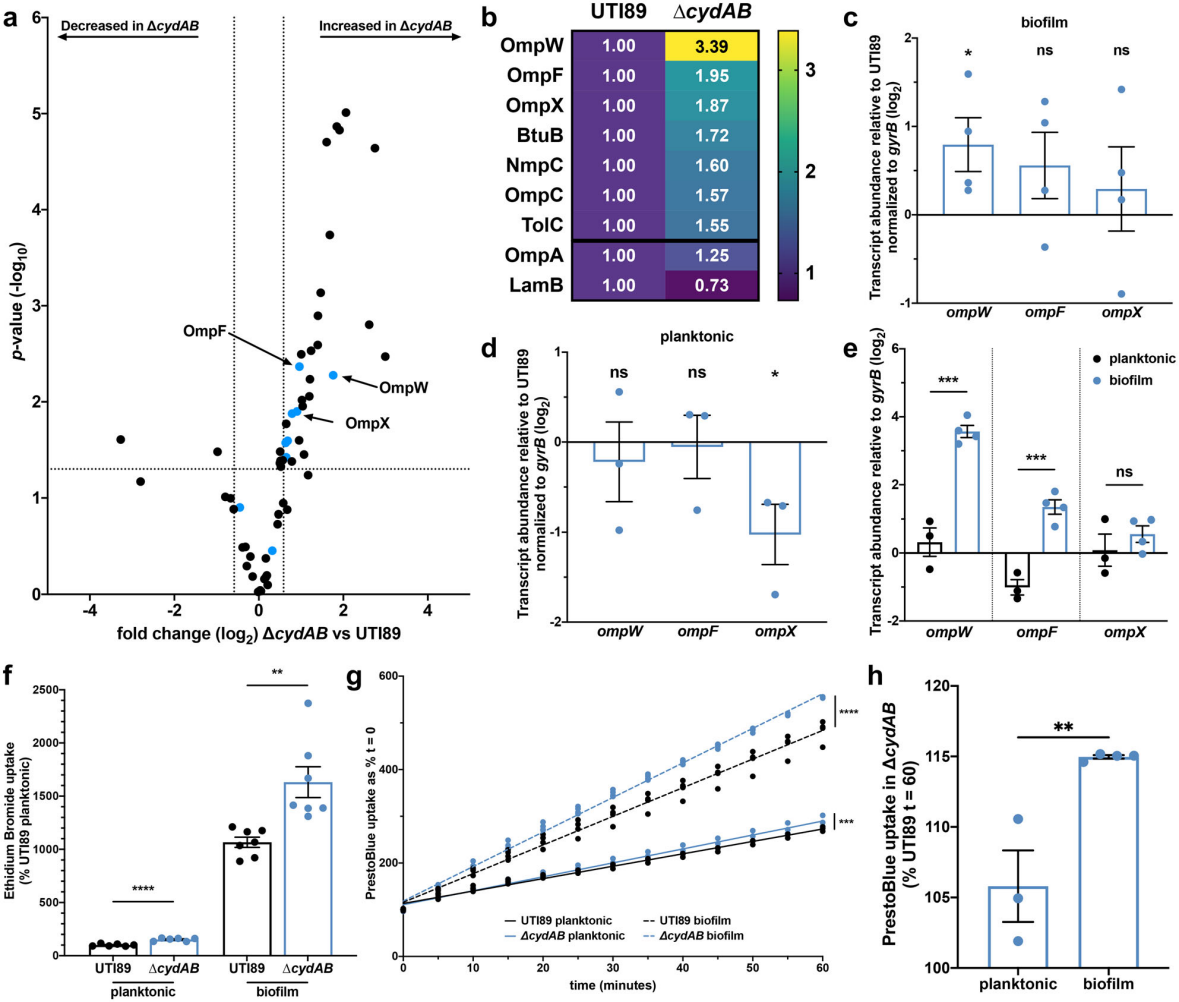
Cytochrome *bd*-deficient biofilm cells have elevated uptake of noxious chemicals. **a** Volcano plot depicting all outer membrane or secreted proteins detected by LC-MS/MS performed on outer membrane and extracellular matrix extracts. Blue dots represent outer membrane channel proteins. **b** Heatmap depicting the relative difference in abundance of outer membrane proteins in UTI89 and ∆*cydAB*. Each cell contains fold difference in abundance relative to UTI89. OmpA and LamB did not attain statistical significance. Data in **a**, **b** are representative of three biological replicates per strain. **c**–**e** RT-qPCR was performed to determine the relative fold difference in outer membrane protein transcript abundance normalized to *gyrB* abundance between UTI89 and ∆*cydAB* in samples derived from homogenized colony biofilms grown on YESCA agar for 11 days (**c**) and planktonic cells (**d**). **e** Difference in outer membrane protein transcript abundance between ∆*cydAB* biofilm and planktonic cells was evaluated by comparing the abundance of each transcript to *gyrB* abundance. Data in **c**–**e** are representative of four biological replicates and were analyzed using a two-tailed unpaired *t*-test. **f** Cellular uptake of membrane impermeant ethidium bromide into planktonic cells and cells extracted from homogenized biofilms. Data are representative of at least six biological replicates and were analyzed by a two-tailed unpaired *t*-test. **g** Cellular uptake of resazurin-based dye PrestoBlue into planktonic cells (solid lines) and cells extracted from homogenized biofilms (dashed lines) was quantified over time. Data were fit to a linear regression model and analyzed by statistically comparing the slope. Data are representative of at least three biological replicates. **h** Percent difference in PrestoBlue uptake at 60 min in ∆*cydAB* cells as compared to UTI89. Data were analyzed with a two-tailed unpaired *t*-test. Except where noted all data are presented as mean ± SEM, and each dot represents a biological replicate. *p < 0.05, **p < 0.01, ***p < 0.001, ****p < 0.0001.

We next sought to investigate how loss of cytochrome *bd* influences outer membrane permeability. To do so, we extracted the outer membrane and extracellular matrix from colony biofilms using established methods and performed liquid chromatography tandem mass spectrometry (LC-MS/MS) (Fig. 5a and Supplementary Table 2)^41^. These experiments identified 30 outer membrane associated or secreted proteins with significantly altered abundance between wild-type and ∆*cydAB* (defined as fold change ≥1.5 and *p* < 0.05) (Fig. 5a and Supplementary Table 2). Of these, two proteins are of significantly decreased abundance in ∆*cydAB* biofilms, and 28 proteins are of significantly increased abundance. Notably, seven of the 28 proteins with increased abundance in ∆*cydAB* are outer membrane channel proteins responsible for the uptake of environmental compounds (Fig. 5a, b, blue dots and Supplementary Table 2), suggesting ∆*cydAB* biofilm cells may have a more permeable outer membrane. In particular, two proteins with significantly elevated abundance—OmpF and OmpC—are classical general diffusion porins responsible for the nonspecific uptake of hydrophilic small molecules including β-lactams, fluoroquinolones, and aminoglycosides^42–44^.

To validate the mass spectrometry results, we performed RT-qPCR on RNA extracted from whole, homogenized colony biofilms. Based on the finding that outer membrane channel proteins are significantly more abundant in ∆*cydAB* biofilms, in combination with the known role for this class of proteins in antibiotic uptake, we chose to measure transcript abundance of the three outer membrane proteins with the most increased abundance in *∆cydAB* (OmpW, OmpF, and OmpX) (Fig. 5b). Consistent with the proteomics data, RT-qPCR revealed that ∆*cydAB* biofilms have significantly more abundant *ompW* transcript (1.9-fold greater than wild-type), and elevated abundance of both *ompF* and *ompX* transcript (1.6 and 1.4-fold greater than wild-type, respectively), albeit below the threshold of significance (Fig. 5c). To determine if the observed increase in *omp* transcript is biofilm specific, we performed RT-qPCR targeting the same genes with RNA derived from planktonic cultures (Fig. 5d). Interestingly, in planktonic cells we observe no significant difference in *ompW* and *ompF* abundance between strains, and significantly decreased *ompX* transcript in ∆*cydAB* (Fig. 5d). Finally, we compared abundance of each transcript between ∆*cydAB* planktonic and biofilm cells and observe that ∆*cydAB* biofilm cells have significantly elevated steady state transcript of *ompW* and *ompF* as compared to ∆*cydAB* planktonic cells (Fig. 5e). These results are in agreement with previous studies in K-12 *E. coli* demonstrating that several outer membrane proteins, including OmpC, OmpF, and NmpC, have elevated expression in biofilms relative to planktonic cultures^45^. Together these results indicate that cytochrome *bd*-deficient cells have elevated expression of several outer membrane proteins in the biofilm state. Guided by these results, we next sought to investigate how loss of cytochrome *bd* influences cellular accumulation of noxious chemicals.

### Cytochrome *bd*-deficient biofilm cells have enhanced uptake of noxious chemicals

Because outer membrane proteins serve as the primary site of cellular entry for hydrophilic small molecules, the increased abundance of outer membrane proteins suggests that ∆*cydAB* biofilm cells may have a more permissive outer membrane, and therefore increased uptake of antibiotics and other noxious chemicals. To test this, we measured accumulation of ethidium bromide into planktonic cells as well as cells extracted from colony biofilms (Fig. 5f). Ethidium bromide is outer membrane impermeant and fluoresces after intercalation into DNA. In planktonic cells we observe a small, but statistically significant increase in ethidium bromide accumulation in ∆*cydAB*. In biofilm cells, ethidium bromide uptake is significantly elevated for both strains and highest in ∆*cydAB* biofilm cells (~10- and 16-fold elevated in UTI89 and ∆*cydAB*, respectively), consistent with the observed increase in outer membrane protein abundance in biofilms (Fig. 5e, f). These data demonstrate that biofilm cells have elevated outer membrane permeability, higher uptake of noxious chemicals as compared to planktonic cells, and that loss of cytochrome *bd* enhances cellular uptake of these compounds.

The increased ethidium bromide accumulation represents a net increase in uptake and could be explained by alterations to the rate of influx, efflux, or both. To differentiate these possibilities, we quantified influx kinetics using resazurin-based PrestoBlue, a dye that becomes fluorescent after import into the cytosol^46^. In planktonic cells we observe a small, but statistically significant increase in the rate of dye influx in ∆*cydAB* relative to wild-type (slope: 2.6 and 3.0 for wild-type and ∆*cydAB*, respectively) (Fig. 5g). Consistent with ethidium bromide uptake data, in biofilm cells we observe a significant increase in the rate of accumulation for both strains (Fig. 5f, g). In addition to the overall increase in influx, we observe a significantly increased rate of influx in ∆*cydAB* biofilm cells as compared to wild-type biofilm cells (slope: 6.1 and 7.4 for wild-type and ∆*cydAB*, respectively) (Fig. 5g). In biofilm cells, the maximal fluorescence value obtained after 60 min was 15 percent higher in ∆*cydAB* compared to wild-type—an ~2.5-fold greater difference than that observed in planktonic cells (six percent) (Fig. 5h). Together these results indicate that biofilm cells have an elevated rate of influx compared to planktonic cells, and that loss of cytochrome *bd* enhances cellular influx of noxious compounds.

### Loss of cytochrome *bd* impairs efflux of noxious chemicals in biofilm cells

**Fig. 6:**
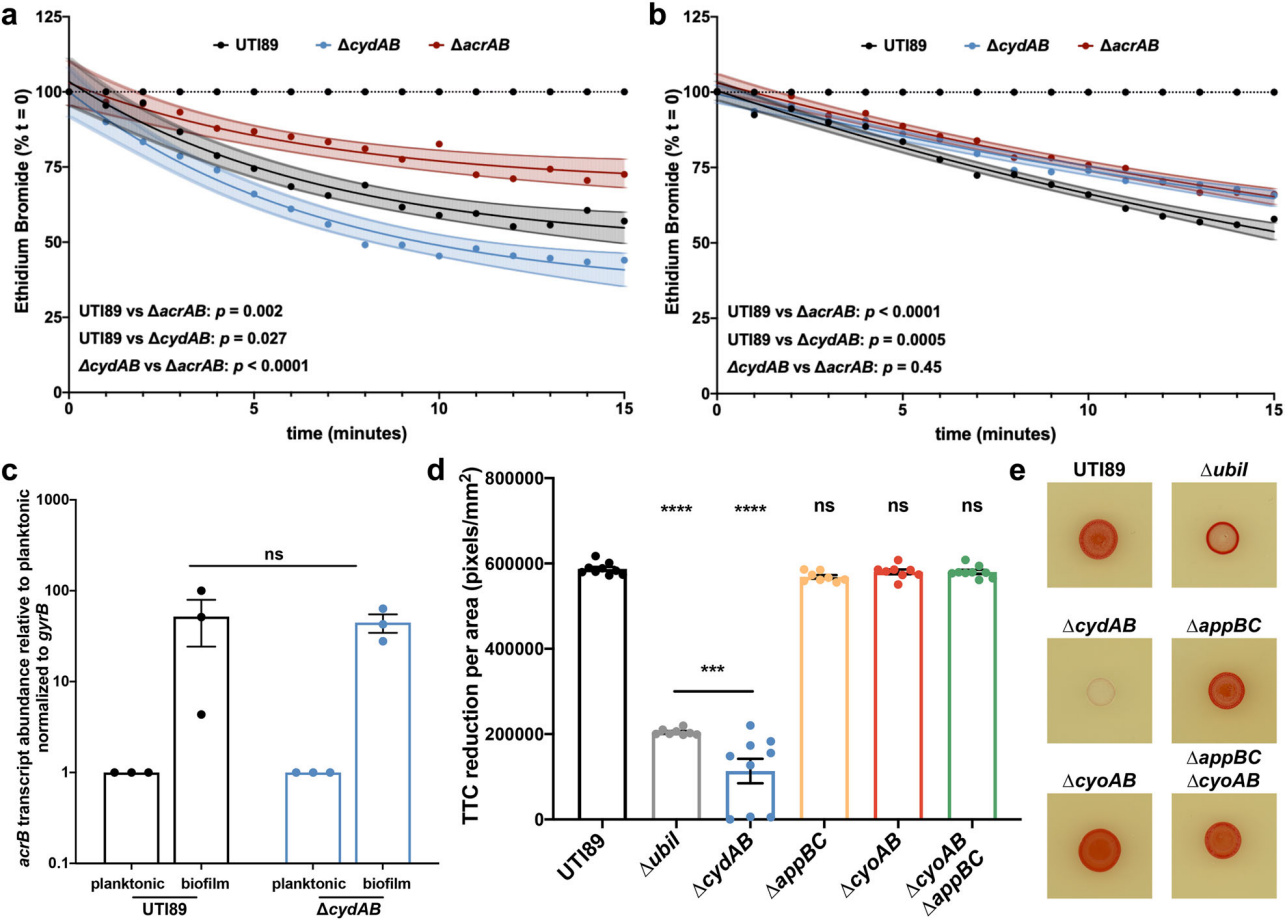
Loss of cytochrome bd impairs efflux by diminishing respiratory flux. **a**, **b** Efflux of ethidium bromide was measured in planktonic cells (**a**) and cells extracted from homogenized colony biofilms (**b**). Data were fit to a one phase decay model, and statistical comparisons were made between the curve of best fit for each strain. Data are presented as mean ± 95 percent confidence interval. **c** RT-qPCR was performed to measure *acrB* transcript abundance normalized to *gyrB* abundance in UTI89 and ∆*cydAB* planktonic and biofilm cells. Mean values were statistically compared with a two-tailed unpaired *t*-test. Data are presented as mean ± SEM, and each dot represents a biological replicate. **d**, **e** Respiratory flux was quantified in wild-type, ∆*ubiI* (ubiquinone synthase mutant), and quinol oxidase mutants by measuring triphenyl tetrazolium chloride (TTC) reduction. **d** Quantification of TTC reduction per unit area in spot colonies. Data were analyzed by a one-way ANOVA with Dunnett’s multiple comparisons test. Data are presented as mean ± SEM, and each dot represents a biological replicate. **e** Representative images of TTC reduction assays. Red color indicates respiratory activity. Data in **a**–**c** are representative of three biological replicates, and data in **d**, **e** are representative of at least eight biological replicates. *p < 0.05, **p < 0.01, ***p < 0.001, ****p < 0.0001.

In addition to affecting the rate of antibiotic influx, loss of cytochrome *bd* may also influence the efficiency of efflux. To test this, we measured the rate of ethidium bromide efflux from wild-type and ∆*cydAB* cells. Cells were loaded with ethidium bromide under energy-limited conditions, and efflux was monitored over time using fluorescence based methods (Fig. 6a, b)^47^. Cells were either left in energy-limited conditions as a control for passive decay in signal, or re-energized by the addition of glucose^47^. The data were fit to a one phase decay model, and differences between strains were determined by statistically comparing the best fit models.

We first measured the rate of efflux in planktonic cells (Fig. 6a). As expected, the slowest decay in signal was observed in a mutant strain lacking *acrAB*, the resistance-nodulation-division (RND) efflux pump primarily responsible for efflux of ethidium bromide (Fig. 6a)^47^. The rate of efflux was significantly elevated in both wild-type and ∆*cydAB* as compared to ∆*acrAB* (UTI89 vs ∆*acrAB*: *p* = 0.002; ∆*cydAB* vs *∆acrAB*: *p* < 0.0001) (Fig. 6a). Surprisingly, the rate of efflux was significantly elevated in ∆*cydAB* as compared to wild-type (*p* = 0.02) (Fig. 6a), indicating loss of cytochrome *bd* enhances efflux under aerobic conditions in planktonic cells.

Next, we homogenized colony biofilms, removed the extracellular matrix, and extracted cells to measure the rate of ethidium bromide efflux in biofilm cells (Fig. 6b). As expected, ∆*acrAB* biofilm cells again have the slowest rate of efflux, and the rate of efflux did not significantly differ between ∆*acrAB* planktonic and biofilm cells (*p* = 0.23) (Fig. 6b). Consistent with data from planktonic cells, we observe a significantly elevated rate of efflux in wild-type biofilm cells as compared to ∆*acrAB* biofilm cells (*p* < 0.0001) (Fig. 6b). The rate of efflux did not significantly differ between wild-type planktonic and biofilm cells (*p* = 0.53). Strikingly, the rate of efflux in ∆*cydAB* biofilm cells was indistinguishable from ∆*acrAB* biofilm cells (*p* = 0.45) (Fig. 6b), indicating that loss of cytochrome *bd* functionally inactivates efflux in biofilm cells. ∆*cydAB* biofilm cells have a significant reduction in efflux both compared to ∆*cydAB* planktonic cells (*p* < 0.0001) and compared to wild-type biofilm cells (*p* = 0.0005). Although ∆*acrAB* biofilms exhibit some minor structural anomalies relative to wild-type, they are morphologically distinct from ∆*cydAB* biofilms, suggesting a lack of AcrAB efflux activity does not fully explain the biofilm developmental defects observed in ∆*cydAB* (Supplementary Fig. 1). These results indicate that loss of cytochrome *bd* impairs efflux of noxious chemicals in a biofilm-specific manner.

### Loss of cytochrome *bd* impairs respiratory flux and diminishes the proton motive force

The impaired efflux in ∆*cydAB* biofilm cells could be explained by changes in the expression, abundance, or activity of efflux pumps. To investigate these possibilities, we performed RT-qPCR on samples derived from planktonic and biofilm cells to measure the abundance of *acrB* transcript. Interestingly, *acrB* transcript is ~50-fold more abundant in biofilm cells relative to planktonic cells, and we observe no significant difference in abundance between wild-type and ∆*cydAB* (Fig. 6c). Additionally, our proteomics results reveal no significant difference in AcrA abundance between strains (fold change 0.99 in UTI89 compared to ∆*cydAB*, *p* = 0.95). These data argue the impaired efflux in ∆*cydAB* biofilm cells is not explainable by changes in *acrAB* expression or abundance; rather, loss of cytochrome *bd* appears to reduce the activity of AcrAB—and potentially other proton dependent tripartitate exporters—in biofilm cells.

Because cytochrome *bd* is a respiratory quinol oxidase that contributes to electron flow and the establishment of the proton motive force, we hypothesized that loss of cytochrome *bd* would impair respiratory flux, diminish the proton motive force, and consequently impair the proton mediated efflux through RND, major facilitator superfamily (MFS), and multidrug and toxin extrusion (MATE) family transporters^48^. To test this hypothesis, we quantified respiratory flux using triphenyl tetrazolium chloride (TTC) reduction assays (Fig. 6d, e). TTC is a redox sensitive dye that undergoes an irreversible color change upon reduction by NADH dehydrogenase and is commonly used as an indicator of respiratory activity^31^. Cells lacking cytochrome *bd* (Δ*cydAB*) displayed significantly diminished overall TTC reduction as compared to wild-type, indicating that loss of cytochrome *bd* diminishes flux through the electron transport chain and impairs the generation of the proton motive force (Fig. 6d, e). Importantly, the diminished TTC reduction in ∆*cydAB* is rescued by extrachromosomal complementation with a plasmid encoding the *cydABX* operon under native transcriptional control (Supplementary Fig. 2)^16^. Despite the observed impairments in respiratory flux, we observe no significant reduction in ATP levels in ∆*cydAB* colony biofilms (Supplementary Fig. 3), consistent with our previous reports^16^. This suggests that these cells likely are not fermenting, as under fermentative conditions the F_0_F_1_-ATPase is reversed and consumes ATP to generate a proton gradient^49^. Rather, the reduced respiratory flux is likely due to inefficient respiration in hypoxic biofilm regions where cytochrome *bd* is most highly expressed^16^. Consistent with this, loss of cytochrome *bo* or *bd*_2_ did not significantly impact TTC reduction, arguing that cytochrome *bd* is the dominant respiratory enzyme under the conditions tested and its loss cannot be compensated for by expression of other quinol oxidases (Fig. 6d, e). Together these data argue that loss of cytochrome *bd* impedes efflux of noxious chemicals by disrupting respiratory flux and impairing the proton dependent activity of efflux pumps.

## Discussion

Living in spatially structured biofilm communities affords resident bacteria significant protection from a wide array of exogenous threats, including antibiotics and host immune defenses. While this has been demonstrated in several different studies, the composite mechanisms that lead to resilience in the biofilm remain largely uncharacterized. In this work, we build upon our previous findings that revealed a spatial organization in quinol oxidase expression in *E. coli* biofilms^16^. Here, we expand our understanding of how cytochrome *bd* expression influences biofilm-specific resistance to antibiotics. We demonstrate that loss of cytochrome *bd* increases antibiotic susceptibility in a biofilm-specific manner by regulating the cellular accumulation of antibiotics and other noxious chemicals. This enhanced accumulation results from a combination of increased abundance of general diffusion porins and decreased efficiency of proton mediated efflux. Consistent with these findings, alterations in the expression or activity of porins and efflux pumps is a common contributor to antibiotic resistance in clinical isolates^42,44,50^.

We report that disrupting cytochrome *bd*-mediated respiration in uropathogenic *E. coli* leads to a general enhancement of antibiotic susceptibility specifically when bacteria are found in the biofilm state. A likely explanation for the distinct antibiotic susceptibility phenotypes observed between ∆*cydAB* planktonic and biofilm cells is that *E. coli* encodes a highly flexible respiratory chain—allowing bacteria to adapt to loss of cytochrome *bd* by altering the expression of other oxidases^17,24^—and that the spatial organization of biofilms imparts unique metabolic constraints not encountered in well-mixed planktonic cultures. Indeed, we previously reported that expression of cytochrome *bo* was increased nearly tenfold in ∆*cydAB* biofilms, indicating that rather than simply impeding respiration, loss of cytochrome *bd* forces bacteria to instead transition to respiring via cytochrome *bo*^16^. Such a transition likely has minimal effect in shaking, logarithmic phase planktonic cultures, where all cells are expected to have access to near atmospheric levels of oxygen, as cytochrome *bo* is a low-affinity respiratory quinol oxidase optimized for use under atmospheric oxygen tensions^17,24^. In fact, because it is a proton pumping oxidase, cytochrome *bo* is more energetically efficient than cytochrome *bd* (H^+^/e^−^ ratio: 2 and 1 for cytochrome *bo* and *bd*, respectively), potentially explaining the increased efflux observed in ∆*cydAB* planktonic cells. In spatially structured biofilms, by contrast, most cells are exposed to subatmospheric oxygen levels^3^. As such, in these cells cytochrome *bd* is the dominant respiratory enzyme, and the low oxygen affinity of cytochrome *bo* ensures that simply overexpressing this oxidase cannot compensate for lack of cytochrome *bd*. As a result of the unique biochemistries of these respiratory oxidases and the spatially structured nature of biofilms, loss of cytochrome *bd* reduces the efficiency of respiration and proton mediated efflux in a biofilm-specific manner.

While our results stand in contrast to the antibiotic resistance phenotype generated by respiratory deficiency in small colony variants described in *Staphylococcus aureus* and other species^51^, they are consistent with studies in *Mycobacterium tuberculosis* in which targeting of the electron transport chain is a promising avenue for the development of novel classes of antibiotics^52,53^. In recent years two electron transport chain inhibitors—bedaquiline, an ATPase inhibitor, and pretomanid, a nitric oxide donor and respiratory poison—have been approved by the Food and Drug Administration (FDA) for the treatment of multidrug resistant *M. tuberculosis*^52,54^. Additionally, promising results from a phase 2 clinical trial were recently reported for telacebec (Q203), an inhibitor of the terminal cytochrome oxidase supercomplex *bc*_*1*_*-aa*_*3*_^55^. In addition to the known clinical utility of these agents, preclinical studies have identified numerous small molecule inhibitors of all electron transport chain components (NADH dehydrogenases, succinate dehydrogenases, quinol oxidases, ATPase), some of which are known to eradicate even highly drug resistant isolates^52,53^. Although the potential for disrupting energetics as a therapeutic approach has not been thoroughly evaluated outside mycobacterial species, in combination with our findings these studies raise the possibility of inhibiting cytochrome *bd* to treat or prevent urinary tract infections. Notably, previous work has identified several natural small molecule quinol analogs that serve as potent inhibitors of *E. coli* quinol oxidases, including several molecules that preferentially inhibit *bd-*type oxidases^52,56^. Importantly, the *bd*-type oxidases are unique to bacteria, suggesting inhibitors of cytochrome *bd* could be used clinically to inhibit biofilm formation and potentiate the effects of antibiotics. Together, this work reveals that the spatial stratification of respiration is a fundamental driver of *E. coli* biofilm stress tolerance and suggest the possibility of rewiring the electron transport chain as an antibiofilm therapeutic approach.

## Methods

### Bacterial strains and growth conditions

All studies were performed in uropathogenic *Escherichia coli* cystitis isolate UTI89^57^ and isogenic deletion mutants. For all analyses, strains were propagated from a single colony in Lysogeny broth (LB) (Fisher) at pH 7.4 overnight at 37 °C with shaking unless otherwise noted. Genetically manipulated strains were created using λ-red recombinase system^58^. Quinol oxidase mutant strains, *ubiI* mutant strains, and ∆*cydAB* complementation constructs were created in previous studies^16,22^. Primers used for gene deletions and qPCR are listed in Supplementary Table 3.

### Crystal violet biofilm assays

Determination of biofilm biomass was performed using the crystal violet assay as previously described^33^. Overnight cultures were diluted to optical density 600 nm (OD_600_) = 0.05, and 100 µL of the diluted culture was aliquoted into a 96-well polyvinyl chloride (PVC) plate (Costar). Plates were incubated in a humid chamber at room temperature, washed and stained with crystal violet, and disaggregated using 35 percent acetic acid. For antibiotic assays, biofilms were grown for 48 h, antibiotics or vehicle was added to each well, and biofilms were grown another 72 h prior to determining biomass. Total biomass was determined by measuring the absorbance at 570 nm using a SpectraMax i3 microplate reader (Molecular Devices).

### Microscopy

Plastic coverslips (Fisher) were placed in the diluted culture in a six well plate and allowed to grow 48 h at room temperature. Antibiotics were added at 48 h, and biofilms were allowed to grow another 72 h before being fixed in 4% PFA and stained with SYTO 9 (ThermoFisher). Images were taken using a Zeiss 710 confocal laser scanning microscope. To obtain a representative sample of the biofilms, at least three images were taken along the air-liquid interface from at least five biological replicates.

### Biofilm survival assays

Colony biofilm survival assays were performed as previously described^35^. Briefly, 10 µL overnight culture was spotted on a piece of filter paper covered in a thin layer of 1.2× yeast extract casamino acids (YESCA) agar and allowed to incubate at room temperature. After 72 h, biofilms were transferred to new YESCA agar with or without antibiotics. After 24 h of treatment, biofilms were homogenized by two rounds of vortexing and sonication, serially diluted, and plated to enumerate colony forming units (CFUs).

### Disk diffusion assays

Antimicrobial susceptibility testing was performed using the Kirby-Bauer disk diffusion method. Testing was performed on 5% Mueller-Hinton agar using commercially available antimicrobial disks (BD) according to Clinical & Laboratory Standard Institute (CLSI) guidelines, M100-ed30^37^. The following disks were used for antimicrobial susceptibility testing: meropenem (10 µg), cefazolin (30 µg), cefepime (30 µg), ceftazidime (30 µg), ceftriaxone (30 µg), aztreonam (30 µg), ampicillin (10 µg), ampicillin-sulbactam (10/10 µg), amoxicillin-clavulanic acid (20/10 µg), piperacillin-tazobactam (100/10 µg), trimethoprim-sulfamethoxazole (1.25/23.75 µg), nitrofurantoin (300 µg), ciprofloxacin (5 µg), levofloxacin (5 µg), amikacin (30 µg), gentamicin (10 µg), tobramycin (10 µg), and tetracycline (30 µg).

### Broth microdilution assays

Broth microdilution assays were performed to determine the minimum inhibitory concentration (MIC), as previously described^59^. Briefly, antibiotics were serially diluted two-fold in a 96-well plate. To increase the precision of the MIC estimate, two independent dilution series were used for each antibiotic. Overnight cultures were diluted to OD = 0.06 in Mueller-Hinton Broth (BD Difco), and 100 µL was added to each well. Cultures were incubated overnight at 37 °C, and MIC was determined by assessing the row at which visible growth of bacteria was inhibited.

### Time kill kinetics assay

Overnight cultures were subcultured in 20 mL Mueller-Hinton Broth (BD Difco) to an OD_600_ = 0.05 and grown 3–4 h to mid-logarithmic phase. Each strain was normalized to an OD_600_ = 0.5 in PBS and split into two flasks. One flask of each strain was inoculated with antibiotic to a final concentration at five times the MIC of wild-type; the other flask served as an untreated control. After addition of the antibiotic, 100 μL of culture was removed from each flask at each time point (0, 15, 30, 60, and 120 min) for CFU enumeration.

### RT-qPCR

RNA was extracted from day 11 colony biofilms or planktonic cultures using the RNeasy kit (Qiagen). RNA was DNase treated using Turbo DNase I (Invitrogen), and reverse transcribed using SuperScript III Reverse Transcriptase (Invitrogen). cDNA was amplified in an Applied Biosystems StepOne Plus Real-Time PCR machine using SYBR green and primers listed in Table S3. All reactions were performed using cDNA from at least three biological replicates. Each reaction was performed in triplicate with at least two different cDNA concentrations. A melt curve analysis was performed using genomic DNA and for every reaction with cDNA to verify primer specificity. Relative fold difference in transcript abundance was determined using the ΔΔC_T_ method^60^. Transcripts were normalized to *gyrB* abundance.

### Cytochrome *c* binding assay

Outer membrane charge was assessed by measuring the amount of cationic cytochrome *c* binding to cells, as previously described^40^. Planktonic cells were extracted from mid-logarithmic phase cultures. Biofilm cells were extracted from homogenized colony biofilms grown for 11 days on YESCA agar. Cells were normalized to OD = 2.0 and washed twice in 20 mM MOPS (pH 7.0). Cationic equine cytochrome *c* (Sigma) was added to 0.5 mg/mL. Cells were incubated with cytochrome *c* for 10 min at room temperature. After incubation, cells were pelleted by centrifugation, and unbound cytochrome *c* was measured from the supernatant by quantifying absorption at 530 nm.

### Proteomics

Outer membrane and extracellular matrix samples were extracted as described previously^41^. Biofilms were grown for 11 days on 1.2× YESCA agar containing 40 µg/mL Congo Red. Biofilms were homogenized in cold 10 mM Tris-HCl pH 7.4 using an Omni Tissue Homogenizer five times for one minute per cycle. To increase yield and robustness, each sample is a pooled collection of 100 individually grown biofilms. Data are representative of three pooled replicates per strain. The homogenate was centrifuged three times for 10 min at 5000 × *g* to remove cells. NaCl was added to the supernatant (final concentration 170 mM) and centrifuged for one hour at 13,000 × *g* to pellet the ECM. The ECM pellet was washed in 10 mM Tris-HCl pH 7.4 with 4% SDS and incubated at room temperature rocking overnight. Twenty five microgram of protein was precipitated by adding 1/3 volume of 100 percent w/v trichloroacetic acid (TCA). After washing two times with ice cold acetone, the protein pellet was resuspended in 8 M urea 100 mM tris pH 8.5, reduced using TCEP, alkylated with iodoacetamide, diluted back to 2 M urea and digested with 0.5 ug of trypsin overnight at 37 °C. Resulting peptides were analyzed by high resolution data dependent LC-MS/MS. Briefly, peptides were autosampled onto a 200 mm by 0.1 mm (Jupiter 3 micron, 300 A), self-packed analytical column coupled directly to a Q-exactive plus mass spectrometer (ThermoFisher) using a nanoelectrospray source and resolved using an aqueous to organic gradient. Both the intact masses (MS) and fragmentation patters (MS/MS) of the peptides were collected in a data dependent manner utilizing dynamic exclusion to maximize depth of proteome coverage. Resulting peptide MS/MS spectral data were searched against the bacterial protein database using MaxQuant-LFQ along with subsequent MS1-based integrations and normalizations^61^. Statistical comparisons of resulting normalized protein quantitative values were performed using ProStaR^62^. Protein name, gene name, and subcellular localization of each identified peptide was manually determined using the UniProt and EcoCyc databases^63,64^.

### Ethidium bromide uptake assay

Colony biofilms were grown at room temperature on YESCA agar. After 11 days, biofilms were homogenized by vortexing and sonication in PBS. After homogenization, a portion of the cellular fraction was removed and normalized to OD_600_ = 0.5 in PBS. Ethidium bromide (Bio-Rad) was added to a final concentration of 10 µg/mL. Cells were then incubated at 37 °C for 10 min. Next, the suspensions were pelleted, supernatant removed, and cells were resuspended in 300 µL PBS. Fluorescence of ethidium bromide was measured at 360/590 nm. Each fluorescence measurement is the average of three technical replicates.

### PrestoBlue uptake assay

Uptake of PrestoBlue (Invitrogen) was performed as previously described^46^. Planktonic cultures were grown to mid-logarithmic phase and normalized to OD_600_ = 1.0 in PBS. Biofilm cells were extracted from homogenized colony biofilms and normalized to OD_600_ = 1.0 in PBS. For each growth condition, 180 µL of culture was mixed with 20 µL PrestoBlue in a 96-well plate. Fluorescence at 560/590 nm was measured every five minutes for 1 h. Each fluorescence measurement is the average of three technical replicates.

### Ethidium bromide efflux assay

Efflux of ethidium bromide was performed as described previously^47^. Planktonic cultures were grown to mid-logarithmic phase, washed twice in PBS, and normalized to OD_600_ = 0.5. Biofilm cells were extracted from homogenized colony biofilms, washed twice with PBS, and normalized to OD_600_ = 0.5. Cells were loaded with ethidium bromide (10 µg/mL) in energy deplete conditions (PBS with 10 µg/mL proton ionophore carbonyl cyanide m-chlorophenyl hydrazone (CCCP)) for one hour at 37 °C. Cells were washed in PBS and resuspended in PBS ± 0.4% w/v glucose. Efflux was monitored by measuring fluorescence at 360/590 nm in technical triplicate at 37 °C every minute for 15 min.

### TTC reduction assays

Triphenyl tetrazolium chloride (TTC) reduction assays were performed as described previously^31^. Ten microliter of overnight culture was spotted onto 1.2× YESCA agar containing 0.001% (w/v) TTC. After 24 h of growth, colonies were imaged using an Epson digital scanner. Images were subjected to automatic thresholding to subtract background, and TTC reduction was quantified by measuring pixel intensity on imageJ. Colony area was determined on Adobe Photoshop.

### ATP quantification

ATP quantification was performed on cells extracted from homogenized colony biofilms grown on YESCA agar for 11 days. One aliquot of biofilm cells was removed, and ATP concentration was determined using the Cell-Glo Titer kit (Promega) according to manufacturer’s protocols. Briefly, 50 μL of bacterial suspension was mixed with an equal volume of Cell-Glo Titer reagent and incubated with shaking at room temperature for 15 min. Luminescence was measured on a SpectraMax i3 plate reader (Molecular Devices) and converted to ATP concentration using a standard curve. A separate aliquot of the same sample was serially diluted for CFU enumeration. To account for differences in the number of cells between samples, ATP concentration was normalized to CFU per biofilm.

### Statistical analyses

Statistical analyses were performed in GraphPad Prism. Details of sample size, test used, error bars, and statistical significance cutoffs are presented in the text or figure legends.

### Reporting summary

Further information on research design is available in the Nature Research Reporting Summary linked to this article.

## Supplementary information

Supplementary Information

Reporting Summary

## Data Availability

Proteomics data have been deposited in the Proteomics Identifications (PRIDE) Database (Accession: PXD023998). All remaining data can be found in the text and supplemental information, or will be made available upon request of the authors.
