# Supplementary material for: Cytochrome bd promotes Escherichia coli biofilm antibiotic tolerance by regulating accumulation of noxious chemicals
Source: NPJ Biofilms Microbiomes. 2021 Apr 16;7:35. doi: 10.1038/s41522-021-00210-x (PMC8052454; doi:10.1038/s41522-021-00210-x)
Supplement: Supplementary file 1 — Supplementary Information [file 41522_2021_210_MOESM1_ESM.pdf]

Supplemental Information For:

Cytochrome *bd* promotes *Escherichia coli* biofilm antibiotic tolerance by regulating accumulation of noxious chemicals

Connor J Beebout,[1] Levy A Sominsky,[2] Allison R Eberly,[1,3] Gerald T Van Horn,[1] and Maria Hadjifrangiskou[1,4]*

[1] Department of Pathology, Microbiology, and Immunology; Vanderbilt University Medical Center; Nashville, TN, USA

[2] Vanderbilt University; Nashville, TN, USA

[3] Current address: Division of Clinical Microbiology, Department of Laboratory Medicine and Pathology; Mayo Clinic; Rochester, MN, USA

[4] Vanderbilt Institute for Infection, Immunology, and Inflammation; Vanderbilt University Medical Center; Nashville, TN, USA

* Address correspondence to: maria.hadjifrangiskou@vumc.org, @BacterialTalk

Antibiotic Name	Antibiotic Class	Mechanism of Action	Breakpoint (mm)	UTI89 (mm)	S/R	Δ*cydAB* (mm)	S/R
meropenem	carbapenem	cell wall inhibitor	16	33.2	S	36.3	S
cefazolin	cephalosporin	cell wall inhibitor	15	26.0	S	28.5	S
cefepime	cephalosporin	cell wall inhibitor	18	38.6	S	41.5	S
ceftazidime	cephalosporin	cell wall inhibitor	21	32.0	S	36.2	S
ceftriaxone	cephalosporin	cell wall inhibitor	23	36.6	S	38.7	S
aztreonam	monobactam	cell wall inhibitor	21	36.7	S	41.2	S
ampicillin	penicillin	cell wall inhibitor	17	21.6	S	22.7	S
ampicillin-sulbactam	penicillin-β lactamase inhibitor	cell wall inhibitor	15	24.7	S	26.2	S
amoxicillin-clavulanic acid	penicillin-β lactamase inhibitor	cell wall inhibitor	18	21.2	S	22.7	S
piperacillin-tazobactam	penicillin-β lactamase inhibitor	cell wall inhibitor	21	30.6	S	31.5	S
sulfamethoxazole-trimethoprim	sulfonamide-dihydrofolate reductase inhibitor	folate biosynthesis inhibitor	16	30.3	S	32.5	S
nitrofurantoin	nitrofuran	reactive cellular damage	17	23.2	S	25.7	S
ciprofloxacin	fluoroquinolone	topoisomerase inhibitor	21	39.6	S	42.0	S
levofloxacin	fluoroquinolone	topoisomerase inhibitor	17	37.0	S	38.2	S
amikacin	aminoglycoside	translation inhibitor	17	24.6	S	24.7	S
gentamicin	aminoglycoside	translation inhibitor	15	25.6	S	26.2	S
tobramycin	aminoglycoside	translation inhibitor	15	24.3	S	24.7	S
tetracycline	tetracycline	translation inhibitor	15	24.3	S	26.5	S

Supplementary Table 1: Disk Diffusion Assays. Disk diffusion assays were performed to measure antibiotic sensitivity in UTI89 and Δ*cydAB.* CLSI breakpoints were used to determine sensitivity versus resistance to each antibiotic. Reported zone of inhibition is the average of at least three biological replicates.

	A	B	C	D	E	F	G	H
1	**UniProt ID**	**Protein Name**	**Gene Name**	**Subcellular Location**	**Fold Change UTI89 vs ∆*cydAB***	**Log2 (Fold Change)**	**P Value**	**Log10 (P Value)**
2	tr|Q1R227|Q1R227_ECOUT	Putative exported protein	UTI89_P011	extracellular space	0.125525147	-2.993951676	0.00337648	2.471535568
3	tr|Q1R8P4|Q1R8P4_ECOUT	Beta-barrel assembly-enhancing protease	*yfgC*	periplasm, membrane	0.148786374	-2.748685687	2.2852E-05	4.641081337
4	tr|Q1RD49|Q1RD49_ECOUT	Penicillin-binding protein activator	*lpoB*	outer membrane	0.163411049	-2.613422558	0.00157157	2.803666525
5	tr|Q1R968|Q1R968_ECOUT	Bifunctional long-chain fatty acids transporter	*fadL*	outer membrane	0.239537481	-2.061676679	9.7553E-06	5.010759345
6	tr|Q1RE62|Q1RE62_ECOUT	Arginine-binding periplasmic protein 1	*artI*	periplasm, membrane	0.266055743	-1.910199551	1.4827E-05	4.828935705
7	tr|Q1R6J1|Q1R6J1_ECOUT	Putative periplasmic protein	*yraP*	periplasm, outer membrane	0.278227955	-1.845660714	1.3626E-05	4.865617625
8	tr|Q1RCH8|Q1RCH8_ECOUT	Outer membrane protein W	*ompW*	outer membrane	0.295345369	-1.759525104	0.0052803	2.277341724
9	tr|Q1R3A8|Q1R3A8_ECOUT	outer membrane lipoprotein Blc	*blc*	outer membrane, cytosol	0.312269546	-1.679136215	0.00018313	3.737233729
10	tr|Q1R8T9|Q1R8T9_ECOUT	uncharacterized lipoprotein	*yfeY*	outer membrane	0.328622813	-1.605495458	1.9771E-05	4.703980148
11	tr|Q1R208|Q1R208_ECOUT	enterotoxin TieB	*senB*	extracellular space	0.362473728	-1.464051661	0.00073195	3.135517179
12	tr|Q1R5B1|Q1R5B1_ECOUT	Starvation induced outer membrane protein	*slp*	outer membrane	0.379379633	-1.398285865	0.00126921	2.896465692
13	tr|Q1RFZ2|Q1RFZ2_ECOUT	Outer membrane lipoprotein	*rcsF*	outer membrane	0.380701995	-1.393265964	0.00255894	2.591939804
14	tr|Q1R1Q3|Q1R1Q3_ECOUT	TraT complement resistance protein	*traT*	outer membrane	0.424135876	-1.237401574	0.00293356	2.532605306
15	tr|Q1RD74|Q1RD74_ECOUT	Flagellar hook-associated protein 3	*flgL*	outer membrane	0.432483284	-1.209283722	0.00580643	2.236090876
16	tr|Q1R8M1|Q1R8M1_ECOUT	Outer membrane protein assembly factor BamB	*yfgL*	outer membrane	0.436351795	-1.196436363	0.00876317	2.057338657
17	tr|Q1REY0|Q1REY0_ECOUT	Ferrienterobactin receptor	*fepA*	outer membrane	0.445534911	-1.166389611	0.05778582	1.238178746
18	sp|Q1RGE3|LPTD_ECOUT	LPS-assembly protein	*lptD*	outer membrane	0.476631728	-1.069053105	0.03527219	1.452567577
19	tr|Q1R572|Q1R572_ECOUT	Glucanase	*yhjM*	extracellular space	0.486676187	-1.03896591	0.0110896	1.955083959
20	tr|Q1RBF4|Q1RBF4_ECOUT	Outer membrane lipoprotein	*slyB*	outer membrane	0.492971306	-1.020424419	0.00957844	2.018705394
21	tr|Q1RCP9|Q1RCP9_ECOUT	Putative outer membrane receptor	*prrA*	outer membrane	0.498638965	-1.003932473	0.00319788	2.495137683
22	tr|Q1RDS8|Q1RDS8_ECOUT	Outer membrane protein F	*ompF*	outer membrane	0.514037016	-0.960055844	0.00429467	2.367070229
23	tr|Q1R7V0|Q1R7V0_ECOUT	Lipoprotein	*nlpD*	outer membrane	0.516877617	-0.952105365	0.02515595	1.599359224
24	tr|Q1REB0|Q1REB0_ECOUT	Outer membrane protein X	*ompX*	outer membrane	0.533727755	-0.905824059	0.0125733	1.900550698
25	sp|Q1R3U1|BTUB_ECOUT	Vitamin B12 transporter	*btuB*	outer membrane	0.579775036	-0.786434879	0.01323518	1.878270114
26	tr|Q1RES9|Q1RES9_ECOUT	Endolytic peptidoglycan transglycosylase	*rlpA*	outer membrane	0.581569816	-0.781975701	0.04173797	1.379468645
27	tr|Q1RCY1|Q1RCY1_ECOUT;tr|Q1RAK8|Q1RAK8_ECOUT	Outer membrane porin protein	*nmpC*	outer membrane	0.623544333	-0.681435958	0.02540088	1.595151319
28	tr|Q1R573|Q1R573_ECOUT	Cellulose synthase operon protein C	*bcsC*	outer membrane	0.627095163	-0.673243703	0.13242154	0.878041367
29	tr|Q1REF8|Q1REF8_ECOUT	Pectinesterase	*ybhC*	outer membrane	0.637639149	-0.649187886	0.01691269	1.771787222
30	tr|Q1R9K0|Q1R9K0_ECOUT;tr|Q1RC18|Q1RC18_ECOUT	Outer membrane protein 1b	*ompC*	outer membrane	0.638605134	-0.647003943	0.03768109	1.423876549
31	tr|Q1R6U8|Q1R6U8_ECOUT	Outer membrane protein	*tolC*	outer membrane	0.643739109	-0.635451977	0.0266908	1.57363837
32	tr|Q1RAF5|Q1RAF5_ECOUT	Putative pesticin receptor	*fyuA*	outer membrane	0.670084888	-0.577584223	0.11315881	0.946311633
33	tr|Q1RAL6|Q1RAL6_ECOUT	Flagellar hook-associated protein 2	*fliD*	outer membrane	0.675164748	-0.566688516	0.04052614	1.392264766
34	tr|Q1REI3|Q1REI3_ECOUT	Peptidoglycan-associated protein	*pal*	outer membrane	0.696262048	-0.522297707	0.04713933	1.32661659
35	tr|Q1RD80|Q1RD80_ECOUT	Flagellar basal body protein	*flgF*	outer membrane	0.700667349	-0.513198427	0.04105226	1.386662971
36	tr|Q1RD75|Q1RD75_ECOUT	Flagellar hook-associated protein 1	*flgK*	outer membrane	0.704544719	-0.505236814	0.03292098	1.482527244
37	tr|Q1R8Q0|Q1R8Q0_ECOUT	Outer membrane protein assembly factor BamC	*nlpB*	outer membrane	0.708268662	-0.497631384	0.04341877	1.362322534
38	tr|Q1R2T5|Q1R2T5_ECOUT	Hemolysin A	*hlyA*	extracellular space	0.723240021	-0.467453583	0.14764304	0.830787033
39	tr|Q1R8N7|Q1R8N7_ECOUT	Polyphosphate kinase	*ppk*	outer membrane, inner membrane	0.73509184	-0.444003588	0.18628135	0.729830624
40	tr|Q1RDQ7|Q1RDQ7_ECOUT	Outer membrane protein A	*ompA*	outer membrane	0.801838419	-0.31861655	0.3514231	0.454169693
41	tr|Q1R3B0|Q1R3B0_ECOUT	Putative toxin of osmotically regulated toxin-antitoxin system	*ecnB*	outer membrane, inner membrane	0.864984147	-0.209254402	0.79934703	0.097264636
42	tr|Q1RBB9|Q1RBB9_ECOUT	Murein lipoprotein	*lpp*	outer membrane, inner membrane, periplasm	0.875936704	-0.191101472	0.63511162	0.197149939
43	tr|Q1RDC4|Q1RDC4_ECOUT	Curli production assembly/transport component	*csgG*	outer membrane, inner membrane, periplasm	0.891002758	-0.166498197	0.66324838	0.178323803
44	sp|Q1RG12|BAMA_ECOUT	Outer membrane protein assembly factor	*bamA*	outer membrane	0.891893486	-0.165056667	0.42241878	0.37425678
45	tr|Q1RBD1|Q1RBD1_ECOUT	Pertactin domain-containing protein	*ydhQ*	outer membrane	0.912285994	-0.132441927	0.69261387	0.159508816
46	tr|Q1R5W6|Q1R5W6_ECOUT	DNA-binding protein HU-alpha	*hupA*	extracellular space, cytosol	0.966763547	-0.04876502	0.92445578	0.034113857
47	tr|Q1R386|Q1R386_ECOUT	Protein HflK	*hflK*	periplasm, inner membrane, cytosol	0.977974497	-0.032131251	0.91034821	0.04079246
48	sp|Q1R7R4|ENO_ECOUT	enolase	*eno*	extracellular space, cytosol	1.010975792	0.015748452	0.94221423	0.025850341
49	tr|Q1REI6|Q1REI6_ECOUT	Membrane spanning protein	*tolA*	outer membrane, inner membrane	1.107158163	0.146861333	0.65309655	0.185022611
50	sp|Q1R6U6|YGIB_ECOUT	UPF0441 protein	*ygiB*	outer membrane	1.148782818	0.200106077	0.40380723	0.393825907
51	tr|Q1R2K0|Q1R2K0_ECOUT	Type 1 fimbriae major subunit FimA	*fimA*	outer membrane	1.217698428	0.284156883	0.50750697	0.294557987
52	tr|Q1R8L2|Q1R8L2_ECOUT	Alpha-2-macroglobulin	*yfhM*	extracellular space	1.245572315	0.316808784	0.3205	0.494171967
53	tr|Q1RD79|Q1RD79_ECOUT	Flagellar basal-body rod protein	*flgG*	outer membrane	1.304408614	0.383395873	0.32475714	0.488441298
54	sp|Q1R3Q0|LAMB_ECOUT	Maltoporin	*lamB*	outer membrane	1.366544176	0.450532098	0.12537694	0.901782342
55	tr|Q1RD81|Q1RD81_ECOUT	Flagellar hook protein	*flgE*	outer membrane	1.510549001	0.595072985	0.13068864	0.883762158
56	tr|Q1RF95|Q1RF95_ECOUT	DNA-binding protein HU-beta, NS1 (HU-1)	*hupB*	extracellular space, cytosol	1.587862307	0.667085813	0.10083737	0.996378469
57	tr|Q1R2V4|Q1R2V4_ECOUT	Putative F17-like fimbrial subunit	UTI89_C4907	outer membrane	1.73218426	0.792592404	0.09736117	1.011614237
58	tr|Q1RAL7|Q1RAL7_ECOUT	Flagellin	*fliC*	outer membrane	1.970314662	0.978426049	0.03304911	1.480840229
59	tr|Q1RDB7|Q1RDB7_ECOUT	Curlin major subunit	*csgA*	outer membrane	6.964677933	2.80005664	0.06761867	1.169933348
60	tr|Q1RDB8|Q1RDB8_ECOUT	Minor curlin subunit	*csgB*	outer membrane	9.63756597	3.26866883	0.02463106	1.608516969

Supplementary Table 2: Mass spectrometry data. Table lists all detected outer membrane or secreted proteins detected by mass spectrometry, fold change in UTI89 relative to ∆*cydAB,* and *p* value. Data are representative of three biological replicates.

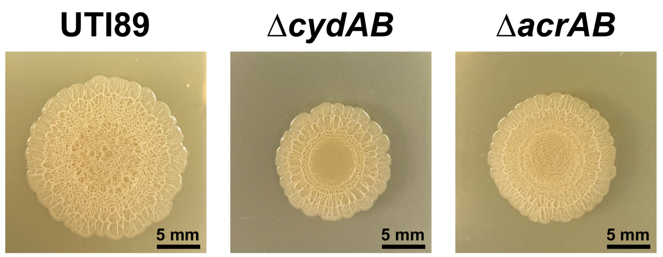

Supplementary Figure 1: AcrAB inactivation has minor effects on biofilm development. Representative images of UTI89, ∆*cydAB*, and ∆*acrAB* biofilms grown on YESCA agar for 11 days. Data are representative of at least five biological replicates.

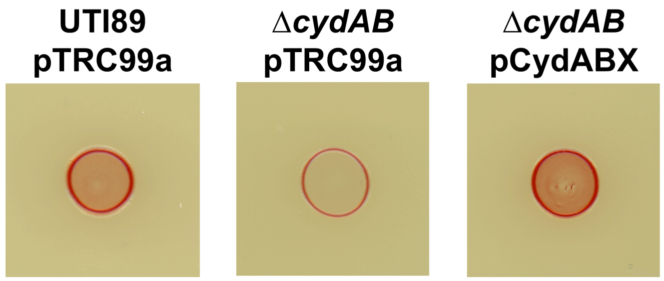

Supplementary Figure 2: Complementation restores TTC reduction in Δ*cydAB*. Representative images of TTC reduction assays. Red color indicates respiratory activity. Strains were transformed with pTRC99a (empty vector) or pCydABX (*cydABX* operon under native transcriptional control). Data are representative of four biological replicates per strain.

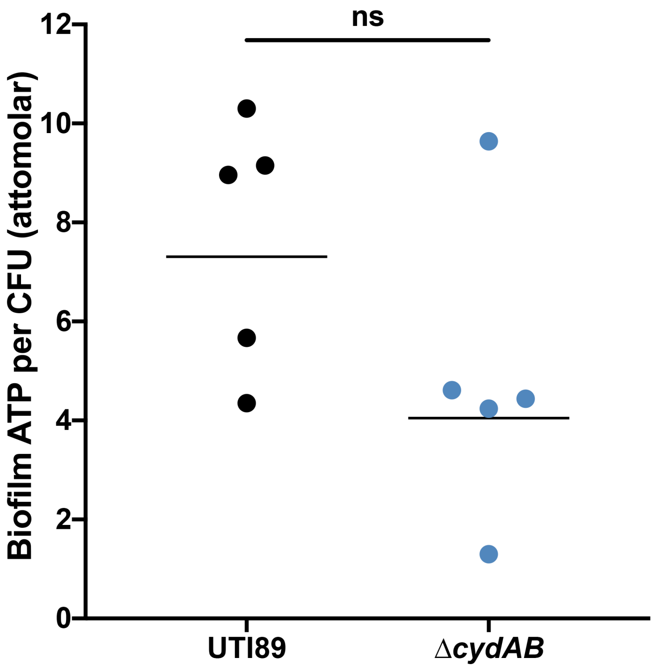

Supplementary Figure 3: Loss of cytochrome *bd* does not impair ATP generation. Graph depicting ATP concentration in colony biofilms normalized to CFUs in the same biofilm. Line depicts geometric mean. Each dot represents a biological replicate. Data were analyzed by a Mann-Whitney test.

Primer	Sequence (5' → 3')	Purpose
acrAB_KO_Fwd	TCAATGATGATCGACAGTATGGCTGTG CTCGATATCTTCATTCTTGCGGCGTGTA GGCTGGAGCTGCTTC	*acrAB* deletion
acrAB_KO_Rev	ATGCCCGCCGTTGGCGTAGTAACAGTC AAAACTGAACCTCTGCAGATCACCATAT GAATATCCTCCTTAG	*acrAB* deletion
acrAB_KOtest_Fwd	TGGTTCAATACTCCTTAATGTTCGTAG	*acrAB* deletion
acrAB_KOtest_Rev	GGCGGTCGTTCTGATGC	*acrAB* deletion
acrB_qPCR_Fwd	GTGTAGTGGTGCGTGCTCT	qPCR
acrB_qPCR_Rev	TATCGTCAGTTCTCCATTACCATTGT	qPCR
ompW_qPCR_Fwd	TATGGCGACCGACAACATTGG	qPCR
ompW_qPCR_Rev	CGTAAGGACGGAATTTGCTGC	qPCR
ompF_qPCR_Fwd	CGTTAGAGCGGCGTGC	qPCR
ompF_qPCR_Rev	CACTGGGTTACACCGATATGCTG	qPCR
ompX_qPCR_Fwd	ACTGGCGGTTACGCACA	qPCR
ompX_qPCR_Rev	CGGACCAGCAGTGATGCC	qPCR

Supplementary Table 3: Primers used in this study.
