# Supplementary material for: Cytochrome bd promotes Escherichia coli biofilm antibiotic tolerance by regulating accumulation of noxious chemicals
Source: NPJ Biofilms Microbiomes. 2021 Apr 16;7:35. doi: 10.1038/s41522-021-00210-x (PMC8052454; doi:10.1038/s41522-021-00210-x)
Supplement: Supplementary file 2 — Reporting Summary [file 41522_2021_210_MOESM2_ESM.pdf]

nature research

Corresponding author(s): Maria Hadjifrangiskou

Last updated by author(s): Feb 4, 2021

Reporting Summary

Nature Research wishes to improve the reproducibility of the work that we publish. This form provides structure for consistency and transparency in reporting. For further information on Nature Research policies, see our Editorial Policies and the Editorial Policy Checklist.

Statistics

For all statistical analyses, confirm that the following items are present in the figure legend, table legend, main text, or Methods section.

n/a	Confirmed	
☐	☒	The exact sample size (*n*) for each experimental group/condition, given as a discrete number and unit of measurement
☐	☒	A statement on whether measurements were taken from distinct samples or whether the same sample was measured repeatedly
☐	☒	The statistical test(s) used AND whether they are one- or two-sided<br>*Only common tests should be described solely by name; describe more complex techniques in the Methods section.*
☐	☒	A description of all covariates tested
☐	☒	A description of any assumptions or corrections, such as tests of normality and adjustment for multiple comparisons
☐	☒	A full description of the statistical parameters including central tendency (e.g. means) or other basic estimates (e.g. regression coefficient) AND variation (e.g. standard deviation) or associated estimates of uncertainty (e.g. confidence intervals)
☐	☒	For null hypothesis testing, the test statistic (e.g. *F*, *t*, *r*) with confidence intervals, effect sizes, degrees of freedom and *P* value noted<br>*Give P values as exact values whenever suitable.*
☒	☐	For Bayesian analysis, information on the choice of priors and Markov chain Monte Carlo settings
☒	☐	For hierarchical and complex designs, identification of the appropriate level for tests and full reporting of outcomes
☒	☐	Estimates of effect sizes (e.g. Cohen's *d*, Pearson's *r*), indicating how they were calculated

Our web collection on statistics for biologists contains articles on many of the points above.

Software and code

Policy information about availability of computer code

Data collection	Data were collected using the following commercially available software: SoftMax Pro 6.4 (Molecular Devices), Zen blue edition (Carl Zeiss Microscopy), Epson Scan v3.9.2.0US (Seiko Epson), StepOne Software v2.3 (Life Technologies).
Data analysis	Data were analyzed using the following commercially available software: Microsoft Excel v16.45 (Microsoft), GraphPad Prism 9.0.0, Adobe Photoshop v13.0 (Adobe Systems), Zen black edition v2.6 (Carl Zeiss Microscopy), ProStaR v 1.22.

For manuscripts utilizing custom algorithms or software that are central to the research but not yet described in published literature, software must be made available to editors and reviewers. We strongly encourage code deposition in a community repository (e.g. GitHub). See the Nature Research guidelines for submitting code & software for further information.

Data

Policy information about availability of data

All manuscripts must include a data availability statement. This statement should provide the following information, where applicable:

- Accession codes, unique identifiers, or web links for publicly available datasets
- A list of figures that have associated raw data
- A description of any restrictions on data availability

Proteomics data has been deposited in the Proteomics Identifications (PRIDE) Database (Accession: PXD023998). All remaining data can be found in the text and supplemental information, or will be made available upon request of the authors.

Field-specific reporting

Please select the one below that is the best fit for your research. If you are not sure, read the appropriate sections before making your selection.

☒ Life sciences ☐ Behavioural & social sciences ☐ Ecological, evolutionary & environmental sciences

For a reference copy of the document with all sections, see nature.com/documents/nr-reporting-summary-flat.pdf

Life sciences study design

All studies must disclose on these points even when the disclosure is negative.

Sample size	All experiments were performed with a sample size of at least three, and sample size was increased where technically feasible. Power analyses were not performed in this study. Sample size is noted in the text and figure legends.
Data exclusions	No data were excluded.
Replication	To ensure reproducibility, all experiments were performed on a minimum of three biological replicates, and multiple technical replicates were included where feasible. This information is noted in the text and figure legends.
Randomization	No randomization was performed in these studies.
Blinding	No blinding was performed in these studies.

Reporting for specific materials, systems and methods

We require information from authors about some types of materials, experimental systems and methods used in many studies. Here, indicate whether each material, system or method listed is relevant to your study. If you are not sure if a list item applies to your research, read the appropriate section before selecting a response.

Materials & experimental systems

n/a	Involved in the study	
☒	☐	Antibodies
☒	☐	Eukaryotic cell lines
☒	☐	Palaeontology and archaeology
☒	☐	Animals and other organisms
☒	☐	Human research participants
☒	☐	Clinical data
☒	☐	Dual use research of concern

Methods

n/a	Involved in the study	
☒	☐	ChIP-seq
☒	☐	Flow cytometry
☒	☐	MRI-based neuroimaging
